# Wear Mechanism Classification Using Artificial Intelligence

**DOI:** 10.3390/ma15072358

**Published:** 2022-03-22

**Authors:** Philipp Maximilian Sieberg, Dzhem Kurtulan, Stefanie Hanke

**Affiliations:** 1 Chair of Mechatronics, Faculty of Engineering, University of Duisburg-Essen, 47051 Duisburg, Germany; 2 Chair of Material Science and Engineering, Faculty of Engineering, University of Duisburg-Essen, 47051 Duisburg, Germany; dzhem.kurtulan@uni-due.de

**Keywords:** artificial intelligence, classification, wear mechanism, sliding wear

## Abstract

Understanding the acting wear mechanisms in many cases is key to predicting lifetime, developing models describing component behavior, or for the improvement of the performance of components under tribological loading. Conventionally scanning electron microscopy (SEM) and sometimes additional analytical techniques are performed in order to analyze wear appearances, i.e., grooves, pittings, surface films, and others. In addition, experience is required in order to draw the correct and relevant conclusions on the acting damage and wear mechanisms from the obtained analytical data. Until now, different types of wear mechanisms are classified by experts examining the damage patterns manually. In addition to this approach based on expert knowledge, the use of artificial intelligence (AI) represents a promising alternative. Here, no expert knowledge is required, instead, the classification is done by a purely data-driven model. In this contribution, artificial neural networks are used to classify the wear mechanisms based on SEM images. In order to obtain optimal performance of the artificial neural network, a hyperparameter optimization is performed in addition. The content of this contribution is the investigation of the feasibility of an AI-based model for the automated classification of wear mechanisms.

## 1. Introduction—Wear Mechanisms

Components whose surfaces make contact and simultaneously perform a sliding relative movement are widespread in technical systems. For example, sliding bearings, cylinders and pistons, ball joints, or forming tools are exposed to such sliding wear. Depending on the type of tribological system—i.e., the materials used, the level and type of load, lubricants, environmental conditions, etc.—different physical and chemical interactions can occur on the contacting surfaces. Various approaches to describe and name these wear mechanisms can be found in the literature. Frequently, mechanisms of wear are categorized into four main types: tribochemical reactions, surface fatigue, abrasion, and adhesion. Under lubricated sliding wear of metals, all of these four main categories can occur, [1].

Surface activation by the mechanical interaction of contacting surfaces in relative motion, as well as the introduced friction, can induce or accelerate chemical reactions. These can result in the formation of non-metallic layers that may break off with ongoing tribological loading. Such a mechanism of wear is categorized and denominated as tribochemical reactions [2].

Surface fatigue is the formation and growth of cracks in or below surfaces in contact, which may lead to the delamination of wear particles. This mechanism is based on the cyclic accumulation of microstructural defects under repeated sliding cycles which lead to the nucleation of cracks [2,3].

Abrasion is the mechanism that occurs most frequently on metals under relative sliding. Here, grooving of one or both surfaces in contact by roughness peaks or particles within the contact leads to the removal of material and the formation of typical grooves and scratches on the surfaces [2,3].

When metal surfaces are in direct contact under high contact pressures, local cold welds may occur. With continuing relative motion, these joints are torn apart and the fracture typically occurs not in the joint interface, but further below the surface in the weaker sliding partner, inducing material transfer from one body to the other. This wear mechanism is denominated adhesion [4].

All wear mechanisms can occur in varying degrees of severity and oftentimes in combination with each other. Consequently, the resulting wear rates can range between ultra-mild wear of <10 nm/h and severe wear of several µm/h or mm/h [5,6].

From a practical point of view, understanding the mechanisms of wear can be highly beneficial for the prediction of changes in performance and the lifetime of technical components. For example, abrasion yields continuous material removal across the complete surface in contact, while surface fatigue typically exhibits an incubation time of crack nucleation without material loss, followed by the detachment of discreet material volumes as delaminating particles [7]. Tribochemical reactions mostly lead to mild wear rates—at least at room temperature—but change the thermal and electrical conductivity across the contact. The frequent rupture of cold-welded joints formed by adhesion may result in high fluctuations of the coefficient of friction and therefore undesired vibrations in the affected technical system—in addition to typically high wear rates [8].

Furthermore, understanding the acting wear mechanisms allows adjusting of the tribological load conditions or the selected materials or surface properties as targeted countermeasures to reduce wear rates. Here, it must be considered that often a combination of different mechanisms act at the same time in different degrees of severity [9]. For example, increasing the hardness of a contacting surface in order to reduce grooving by abrasion [10] may in some cases increase wear by surface fatigue, since an increase in brittleness may accelerate crack propagation [7].

## 2. Wear Mechanism Classification—State of the Art and Future Potential of AI

**Figure 1:**
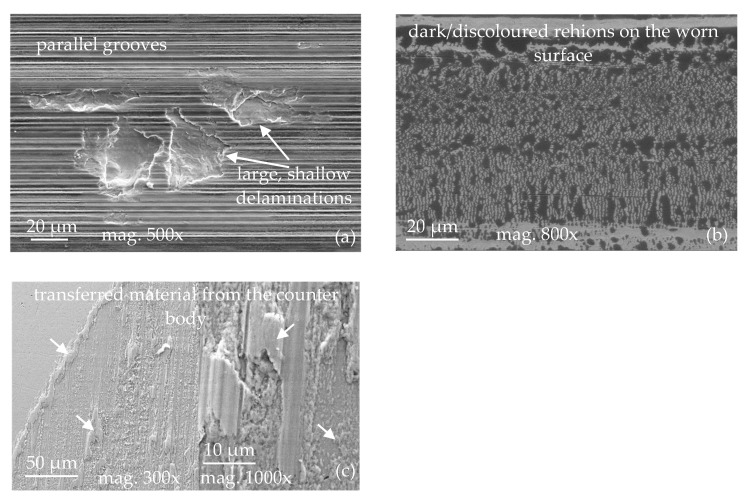
SEM images displaying typical wear appearances on steel surfaces after lubricated sliding wear. (**a**) Abrasion and surface fatigue, (**b**) Tribochemical reactions, and (**c**) material transfer by adhesion.

Commonly, wear mechanisms are classified using ex situ microscopy of the surfaces that were previously in contact. Scientific publications and guidelines published by technical societies are available to support practitioners in identifying wear mechanisms [11,12,13]. Still, expert knowledge and experience are required to understand the mechanisms from the features and wear appearances in the obtained microscopy images reliably. In Figure 1, examples of very typical wear appearances observed by scanning electron microscopy are shown. The wear appearances on Figure 1a consist of long, parallel straight grooves, which clearly indicate the mechanism of abrasion. Additionally, large but shallow pits are visible, where the material has delaminated from the loaded surface. These are typical for surface fatigue, which obviously acts at the same time as the abrasive mechanism. Such delaminations as shown in the figure may be classified as “pitting wear” or “spalling” if occurring, e.g., on bearings or gears, since those are common terms in these technical applications [13]. In order to limit the number of classes in this study, all types of fatigue-type damage are summarized in the class “surface fatigue”. In Figure 1b, the most striking feature is the structures of darker appearance on the loaded surface. Samples are generally cleaned thoroughly using solvents suitable for the applied lubricants before being inserted into the vacuum chamber of the SEM. Any volatile substances remaining on the samples could evaporate under vacuum and contaminate the microscope. Such dark surface regions are typical appearances of tribochemical reactions taking place at room temperature wear. Reaction products contain high amounts of oxygen, carbon, and elements from the lubricant, and therefore appear in different grey shading than the metallic surface in the SEM image. These reaction products are incorporated into the surface material or are chemically bonded to the surface, and therefore remain after cleaning with a solvent. The distribution and appearance of such tribochemical reaction layers may vary significantly, depending on the load conditions, materials, and lubricants from which they are formed. In Figure 1c, two images recorded at different magnifications on the same worn surface are shown. Here, adhesion has occurred, and material adhering on top of the body’s surface is recognizable. Observing only the high magnification image on the right-hand side, this fact cannot be concluded easily, but the shape and structure of the surface appearance visible at the lower magnification strongly support this conclusion. Oftentimes, images at several magnifications are taken, revealing different aspects of the wear appearances, and the conclusions drawn by experts rely on their combined evaluation.

It must be emphasized here that this chapter refers to the evaluation of wear appearances by SEM only. Particularly in the case of material transfer, it is appropriate to conduct additional chemical analyses in order to provide proof for the adhesion of foreign material to a surface. Other measurement techniques, including 3D surface topography analysis, chemical composition measurements of thin surface films, etc., can be employed to analyze wear appearances in more detail. Still, high-quality SEM images of the wear appearances are the most efficient and conclusive source of information and allow to conclude on the acting wear mechanisms in most cases at high reliability—if evaluated by an experienced expert.

In addition to this approach based on expert knowledge, the use of artificial intelligence presents a promising alternative for determining wear mechanisms. Here, one possible approach for implementing the classification is based on the domain of machine learning. Machine learning is a subfield of artificial intelligence [14]. Many different approaches exist in the domain of machine learning. Among them are decision trees [15], probabilistic models such as Bayesian networks [16], support vector machines [17], and artificial neural networks [18]. The focus of the classification method within this contribution is on artificial neural networks, as these are a proven method [19]. Basically, there are different types of artificial neural networks, such as recurrent neural networks [20] and convolutional neural networks [21]. In the following, the focus is limited to convolutional neural networks, as these represent the state-of-the-art network type in image classification [22]. This type of artificial neural network applies convolutions to the input image through multiple trained filters to extract features. By sequentially arranging convolutions, more complex features can be identified. Convolution neural networks thus allow learning of complex features that enable classification [23].

In the following, the focus is on AI-based classification of wear mechanisms. Whereas SEM images have not been used as a foundation for the classification of wear mechanisms before, there are studies on the use of AI for the classification of wear particles for the monitoring of the wear state of machinery. In [24], a multilayer perceptron is used to classify wear particles based on the morphological attributes such as the size and aspect ratio. This allows the underlying wear process to be deduced without expert knowledge by using an artificial neural network. The authors of [25] use a convolutional neural network to evaluate ferrograph images to assess the prevailing wear situation, particularly in terms of severity. Oil samples containing the wear particles are taken from different pieces of machinery from mining and petrochemical industries. The images of the wear particles are classified in terms of initial wear, normal wear, abnormal wear, and severe wear. In addition to the evaluation of wear particles, the use of AI is also investigated for the identification of tool wear. In [26], a deep learning approach is used in combination with transfer learning to accomplish a tool wear classification with respect to classes “thermal wear”, “adhesion”, “chip”, and “notch”. Here, a fine-tuned convolutional neural network achieves a better performance than a custom approach based on a convolutional neural network combined with a support vector machine. For the real-time determination of tool wear on the basis of machine vibrations and photographs of the tool’s cutting edge, ref. [27] combine a convolutional neural network with an artificial neural network based on long short-term memory cells. The convolutional neural network is used to extract features, which are then put into a temporal context by the artificial neural network based on the long short-term memory cells. The classification task includes the classes “initial wear”, “normal wear”, and “rapid wear”. By combining the two different artificial neural network topologies, real-time monitoring of the tool wear state was successfully performed.

To the best of our knowledge, no attempt has been published to carry out a fundamental categorization of the basic wear mechanisms on the surfaces of various tribological systems on the base of SEM images using AI. In the present contribution, SEM images recorded in previous studies during several years were gathered and labeled according to the observable wear mechanisms. They all stem from sliding wear tests in ball-on-plane configuration from a laboratory tribometer, and tribosystems consist of different material combinations and lubricants. Since typically several wear mechanisms act at the same time and are visible on a single SEM image, for the initial investigation surface fatigue was chosen to be identified on the images, in relation to images not displaying signs of surface fatigue. By this initial work, we determine the extent to which the research gap for classifying wear mechanisms based on SEM images can be closed by artificial intelligence.

## 3. Materials and Methods

Within the following section, the development of the artificial neural network is presented for the classification of wear mechanisms. For this purpose, the underlying database and the experimental procedure for its creation are considered first in Section 3.1. Subsequently, the focus is on the implementation of the artificial neural network. Here, a hyperparameter optimization is initially presented in Section 3.2, before the final training of the artificial neural network is described in Section 3.3.

### 3.1. Database

In this contribution, SEM images of the worn surfaces of three different tribological systems are used to train an artificial neural network to recognize the wear appearances. This subsection describes the database used for the training, and in detail the procedure of wear tests, the used materials, and further data preprocessing.

#### 3.1.1. Tribological System

Square flat specimens measuring 11 mm × 11 mm × 4 mm were used as base bodies. Three different material combinations were used for the wear tests by altering the base body material. One was a ferritic–pearlitic steel typically used for freight train wheels, denominated ER7 (DIN EN 13262:2010-07). Furthermore, a thermomechanically modified tool steel with a very fine and homogeneous martensitic microstructure for applications in hot forging was tested, with the trade name CP4M (Dörrenberg Edelstahl GmbH, Engelskirchen-Ruenderoth, Germany). The third material was a Cr-base alloy denominated Cr60Ni40 (Klaus Kuhn Edelstahlgießerei GmbH, Radevormwald, Germany), which is a cast alloy used for high temperature, high wear applications, e.g., in large diesel engines. All base body specimens were ground with sandpaper of increasingly finer grade, up to 1200 grit.

Only one type of counter body was used on the three different base body materials. The counter body of each test was a standard bearing ball (grade G20) made from 100Cr6 (1.3505), with a nominal diameter of 10 mm. According to DIN EB 5401, these have a maximum dimensional deviation of 0.7 µm and a hardness between 60 to 66 HRC. All counter bodies are deployed in as-delivered condition, corresponding to a polished surface. The chemical composition of all materials can be found in Table 1.

#### 3.1.2. Experimental Setup and Microscopy

**Figure 2:**
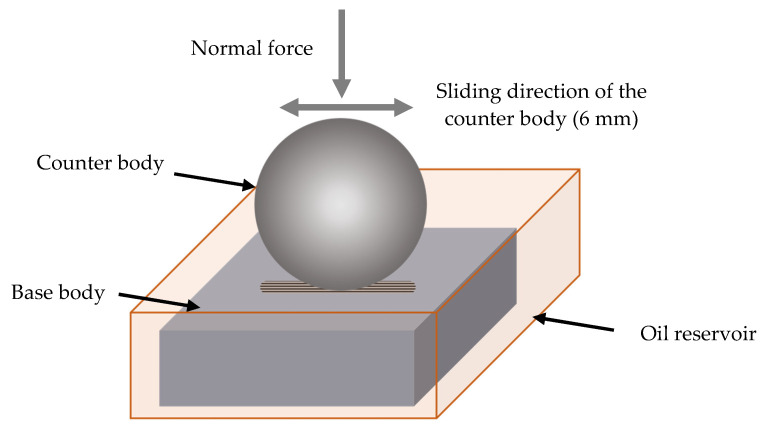
Schematic of the experimental test setup.

Reciprocating sliding in a ball-on-flat configuration was applied to all material combinations on a custom-built tribometer based on a Tytron 250 (MTS System Corporation, Eden Prairie, MN, USA) test system. Figure 2 shows a schematic detail of the test setup, displaying both base and counter body, submerged in the lubricant, and the main tribological loads.

The base body was attached via the specimen holder, which also contains a cutout as an oil reservoir for the lubrication of the tribosystem. Anti-corrosion oil Anticorit PL 3802 39 S 8 (Fuchs GmbH, Mannheim, Germany) with a viscosity of 60 cSt at 60 °C, and silicon oil (Carl Roth GmbH, Karlsruhe, Germany) with a viscosity of 50 cSt were used for the lubrication of the tribosystems.

The holder for the base body is clamped onto a force gauge plate (Kistler Instrumente GmbH, Sindelfingen, Germany) by which normal and frictional force are recorded. The counter body holder was used for the application of a constant normal force by dead weights, varying between 20 N and 180 N. The stroke of the reciprocating sliding movement was 6 mm. The number of cycles was varied between 12,500 and 1,000,000 cycles, with a frequency of 5 Hz and an average sliding speed of 0.06 ms^−1^. An overview of the test parameters for each material combination can be found in Table 2.

After each test, any residual oil on the surfaces of the base and counter body was removed by ultrasonic cleaning using petroleum ether and ethanol as solvents. After cleaning, the specimens’ wear appearances are investigated with a scanning electron microscope (Leo Gemini 1530, Zeiss, Oberkochen, Germany).

#### 3.1.3. Data Preprocessing

A total of 778 images result from the experiments with this test setup. These are classified by expert knowledge with regard to the wear mechanism surface fatigue. This ultimately results in two classes as the basis for the classification. The class “surface fatigue” comprises 242 images while the second class “other wear mechanisms” includes 536 images. About 70% of the data available in the classes are used for training and about 15% each as validation and test data. This leads to the result that for the class “surface fatigue”, 170 images are allocated for training, 36 images for validation, and 36 images for testing. In contrast, for the class “other wear mechanisms” 384 images are provided for training and 76 images each for validation and testing. On this basis, there are thus significantly fewer images available for the implementation of the artificial neural network for the class “surface fatigue” than for the class “other wear mechanisms”. Such a class imbalance will directly affect the achievable classification accuracy [28]. Besides algorithm-level approaches to counteract this class imbalance, further data-level approaches exist [29]. A simple data-level method is oversampling [30]. Here, images are randomly duplicated from the respective datasets. After oversampling, there are also 384 images available for training and 76 images each for validation and testing with respect to the class “surface fatigue”. Table 3 summarizes the database for the implementation of the classifier based on artificial intelligence.

This limitation of available data is also the reason why no further detailed classification of wear mechanisms was carried out. If selecting more precise submechanisms of wear for classification, e.g., “spalling” as described in [13], the number of images displaying this mechanism would be even lower and the class imbalance even more pronounced. The present work, therefore, aims to initially evaluate the potential of AI to differentiate the main mechanisms of wear based on a limited, pre-existing dataset not prepared specifically for this task.

### 3.2. Hyperparameter Optimization

A hyperparameter optimization is performed to achieve an optimal design of the artificial neural network for the classification of the surface fatigue wear mechanism. The algorithm applied belongs to the sequential model-based optimization, which is among the informed model-based optimization methods [31]. These methods are superior to uninformed algorithms, such as random search [32].

Here, a probabilistic model characterizes the objective function of the optimization. Based on the previously evaluated hyperparameter configurations and further available information, the probabilistic model is generated and updated. To select the next hyperparameter configuration, an acquisition function is utilized, determining the predictive distribution of the probabilistic model. By maximizing the acquisition function over the search space, the optimal hyperparameter configuration is determined, [31].

The hyperparameter optimization, as well as the training, are done in Python using the open-source deep-learning library Keras v2.2.4 [33]. The data flow-oriented framework TensorFlow v1.12.0 is used as a backend [34]. Within this contribution, the sequential model-based optimization is conducted by using the Python library Hyperopt v0.2.3 [35]. The acquisition function is represented by the expected improvement [36]. Here, the tree-structured Parzen estimator is applied [37].

Within the optimization, the focus is on the parameters of the convolutional layers. The optimization algorithm Adam is used for the training of the individual artificial neural network configurations, where each configuration is trained for 25 epochs [38]. The evaluation metric used is the binary cross entropy. The fixed hyperparameters are listed in Table 4.

In the sequential model-based optimization, among other things, the number of convolutional layers and the learning rate are adapted. Furthermore, the filter and kernel size of each convolutional layer are optimized. The hyperparameter optimization is performed on a reduced dataset. Table 5 summarizes the search space.

The results of the hyperparameter optimization are shown in Figure 1. Here, the accuracy is chosen as the representation metrics. The accuracy determines the fraction of predictions, which the respective artificial neural network correctly matched:

(1) $$\mathrm{Accuracy}\;=\frac{\mathrm{Number}\;\mathrm{of}\;\mathrm{correct}\;\mathrm{predictions}}{\mathrm{Number}\;\mathrm{of}\;\mathrm{predictions}}.$$

**Figure 3:**
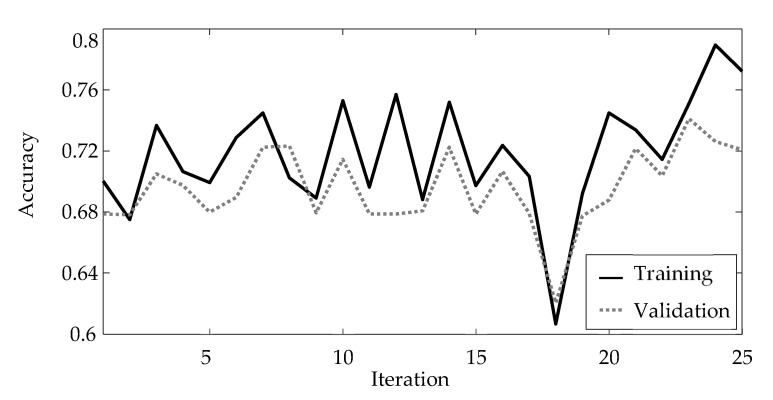
Results of the hyperparameter optimization.

Both the accuracy based on the training and the validation data are displayed in Figure 3. A solid black line represents the accuracy determined for the training data, whereas a dotted grey line illustrates the accuracy for the validation data. The highest accuracy with respect to the training data is achieved in iteration 24 of the hyperparameter optimization with an accuracy of 0.7895. The corresponding accuracy for the validation data for this iteration equals 0.7263.

For training of the artificial neural network, Section 3.3, the hyperparameters from this 24th iteration are used. Table 6 summarizes these hyperparameters and thus highlights the result of the hyperparameter optimization.

**Figure 4:**
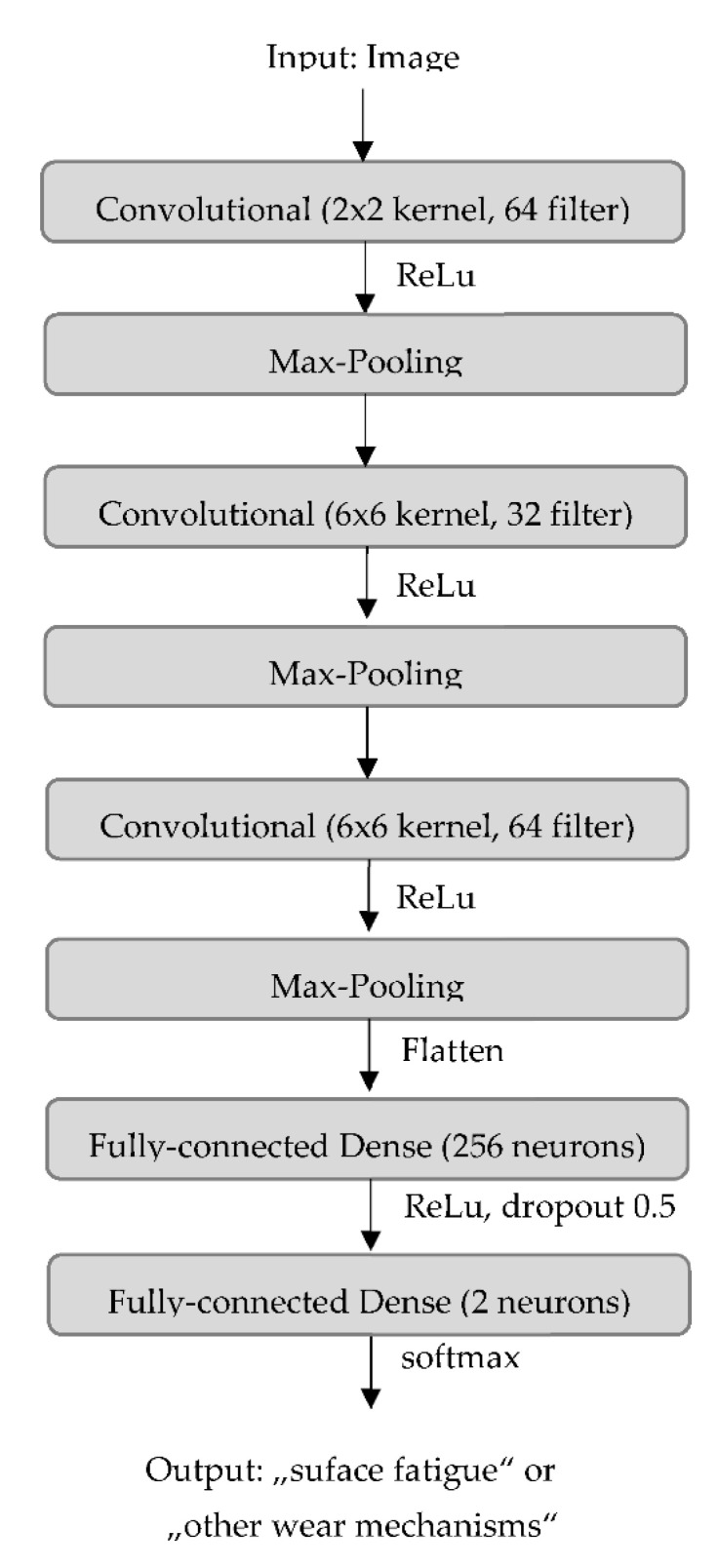
Structure of the artificial neural network for the classification.

The complete structure of the artificial neural network resulting from the hyperparameter optimization is illustrated in Figure 4.

### 3.3. Training

The final training of the artificial neural network follows the hyperparameter optimization. For this purpose, the hyperparameters determined by the optimization are used as the parameter configuration of the classifier based on the artificial neural network. The classifier is trained with 768 images, representing both classes equally. The validation data consists of 152 images, which are also equally divided between both classes. A total of 152 images are reserved for testing and remain unseen during training.

**Figure 5:**
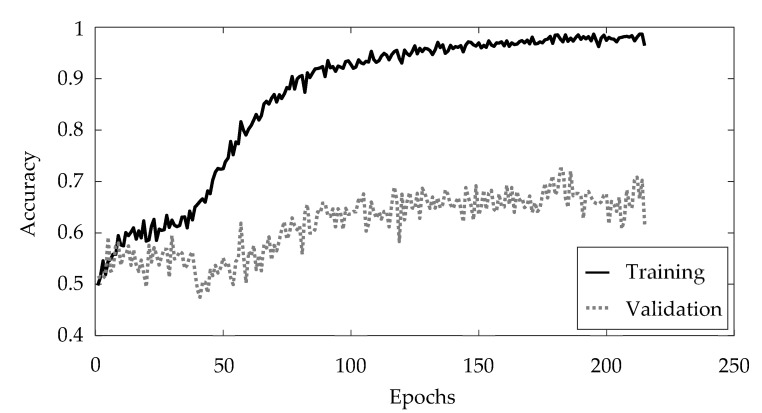
Training results.

The training is performed for a maximum of 250 epochs. To prevent overfitting, early stopping is used [39]. Within this contribution, the training is stopped as soon as the accuracy on the validation data set has not improved for a number of 30 epochs. The courses of the accuracy for the training data and the validation data, respectively, are illustrated in Figure 5. The color scheme regarding the training data and the validation data remains consistent.

**Figure 6:**
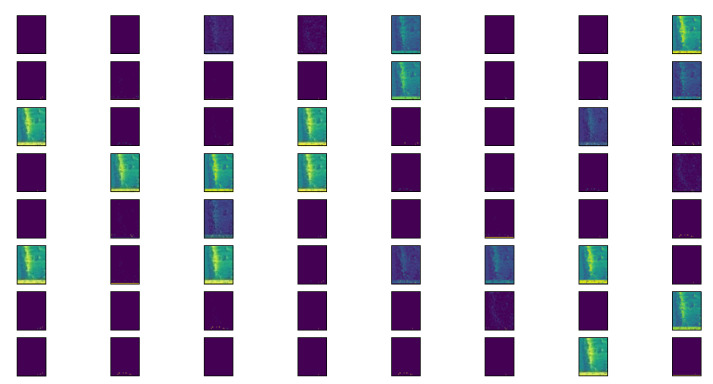
Feature maps of the artificial neural network.

The training is stopped at epoch 215 due to the early stopping option, since the classification accuracy for the validation dataset did not improve from epoch 185 onward. The artificial neural network achieved an accuracy of 97.89% for the training data and 71.96% for the validation data. Figure 6 shows an overview of the features generated by the first convolutional layer, yielding a total of 64 feature maps.

## 4. Results and Discussion

In order to evaluate the performance of the classification by the trained artificial neural network, a dataset unseen during training is used. This test data set consists of 152 images evenly distributed. Both the class “surface fatigue” and the class “other wear mechanism” are represented by 76 images each, whereby a database augmentation has taken place for class “surface fatigue”.

**Figure 7:**
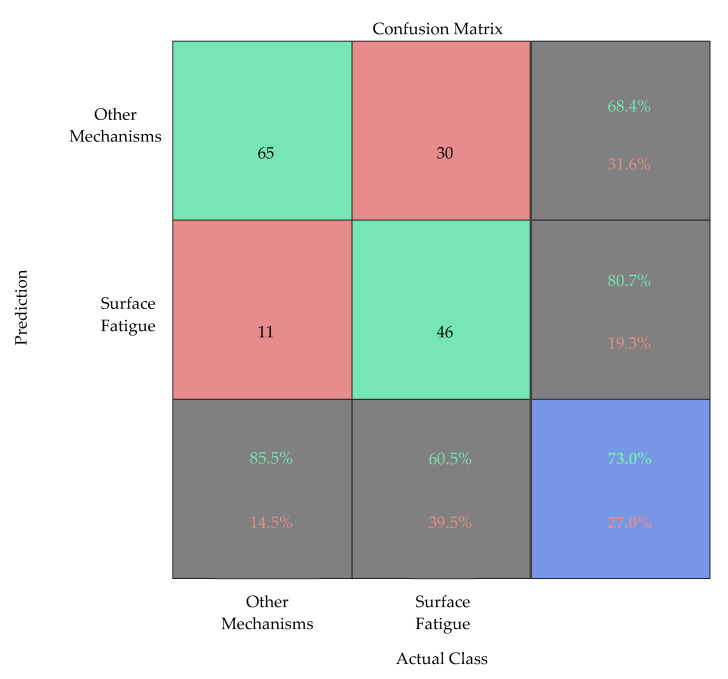
Testing results—confusion matrix.

For the test dataset, the artificial neural network demonstrates an overall classification accuracy of 73%. Thereby, a correct classification of the images of the class “other wear mechanism” is achieved for 65 images. Consequently, 11 images of this class are incorrectly classified. The classification accuracy for this class is thus about 85.5%. The classification of the images of the class “surface fatigue” is correct for about 60.5%. In total, the artificial neural network correctly classifies 46 of the 76 images. An overview of the classification performance is shown in Figure 7 by a confusion matrix.

Overall, the classification accuracy with respect to the test dataset is thus in a similar value range as that for the validation dataset. Basically, however, there are different classification accuracies with respect to the different classes. Whereas the class “other wear mechanisms” is identified with an accuracy of 85.5%, there is merely a classification accuracy of 60.5% on the test data for the class “surface fatigue”. One reason for this lack of accuracy is the problem of the database, which has already been mentioned in Section 3.1. The original database showed an imbalanced class distribution. In this case, 536 images belonged to the class “other wear mechanisms”, whereas only 242 images related to the class “surface fatigue” were available.

To counteract this imbalance, the data of the class “surface fatigue” were augmented by oversampling [40]. With regard to the classification of wear mechanisms, especially surface fatigue, the issue of class imbalance is only partially addressed by oversampling. Since no new information regarding surface fatigue is generated by this approach, the classification accuracy still has potential for improvement. However, the presented classification approach based on artificial intelligence shows an overall satisfactory result. By taking into account additional data, which not only solves the issue of unbalanced classes but also fundamentally increases the amount of available data, further improvements in the classification accuracy are expected [41].

In the following, two correct classifications per class and two incorrect classifications are considered in detail regarding the test dataset.

**Figure 8:**
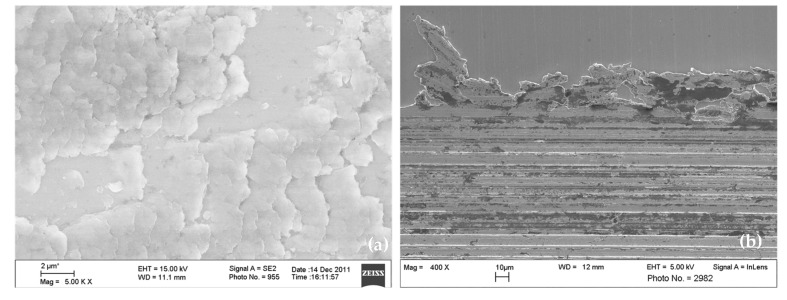
Correct classifications—“other wear mechanisms”. (**a**) Material transfer by adhesion, (**b**) edge of a wear track displaying grooves by abrasion, dark regions from tribochemical reactions, and deformed material pushed out of the contact.

In Figure 8a, appearances of material transfer by adhesion are recognizable by scale-shaped structures on the surface. This is one typical form of material transfer, in which the sliding direction of the counter body can be deduced from the shape of the scales, in this case from left to right in the image. The low contrast of the image, which is obviously caught by the human eye, is not decisive for the automatic classification. Figure 8b displays the edge of a sliding wear track, with the sliding direction oriented horizontally in this image, visible by the grooves from abrasion. Furthermore, dark areas containing tribochemical reaction products can be observed. Material has been pushed out of the contact by plastic deformation, pieces of which are still hanging onto the edge of the wear track. For the human expert, no signs of surface fatigue are visible here, and the image was also correctly classified by the artificial neural network.

**Figure 9:**
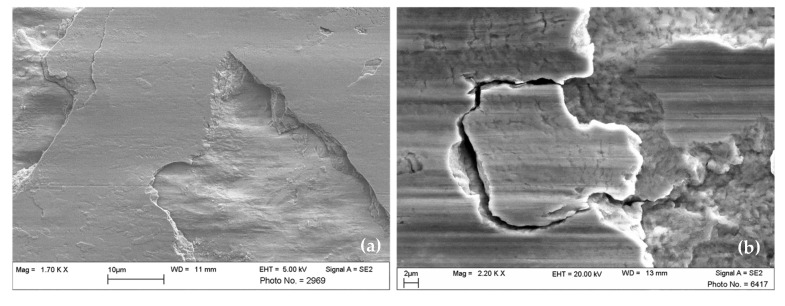
Correct classifications—“surface fatigue”. (**a**) Detail of large delaminations with smooth fracture edges; (**b**) detail of smaller delaminations with rough fracture surface, also displaying a crack and a piece of material not yet detached.

Examples for correctly classified images displaying appearances of surface fatigue are presented in Figure 9. In Figure 9a, a detail of very large delaminations with smooth edges and fracture surfaces is visible, while Figure 9b shows a detail of smaller delaminations with rough, uneven edges and fracture features. Additionally, cracks are visible in both images—particularly obvious in (b).

**Figure 10:**
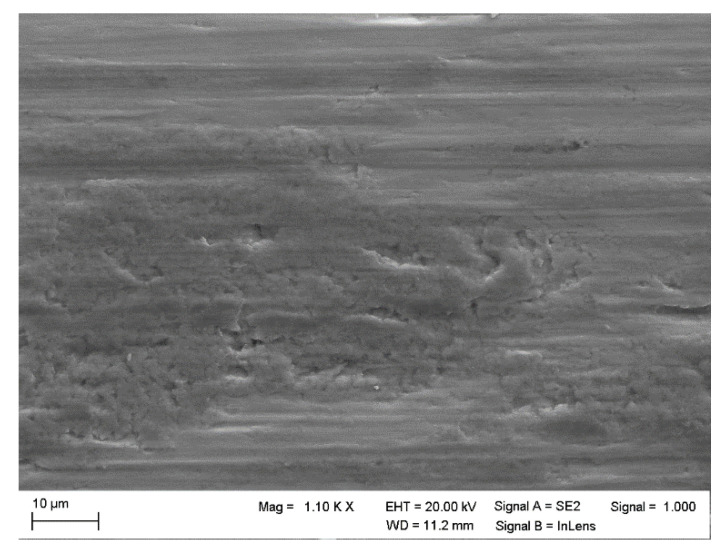
False classification—“other wear mechanism”. Wear debris or transferred material from the counter body containing voids and breakouts are recognizable to the expert.

Furthermore, two incorrect classifications, one for each class, are examined in detail. First, an incorrect classification of the class “other wear mechanisms” is considered. The corresponding image is shown in Figure 10. The artificial neural network incorrectly assigns this image to the class “surface fatigue”. Manual expert analysis during labeling had assigned this image to “other wear mechanisms” because it displays wear debris or transferred material adhering to the surface, which locally contains voids or breakouts that have formed with ongoing sliding during wear testing. In terms of the pattern of these breakouts, the existing image thus resembles the images from the class “surface fatigue”. Expert analysis recognizes this difference based on differences in grey shading and structure of the adhering material, and by relying on additional images from the same sample at different magnification. One possibility to counteract this incorrect classification is to extend the classification task by additional classes for future applications. Thus, apparently similar images, which, however, result from different wear mechanisms, can be unambiguously classified. At the same time, this approach would additionally counteract the existing class imbalance.

**Figure 11:**
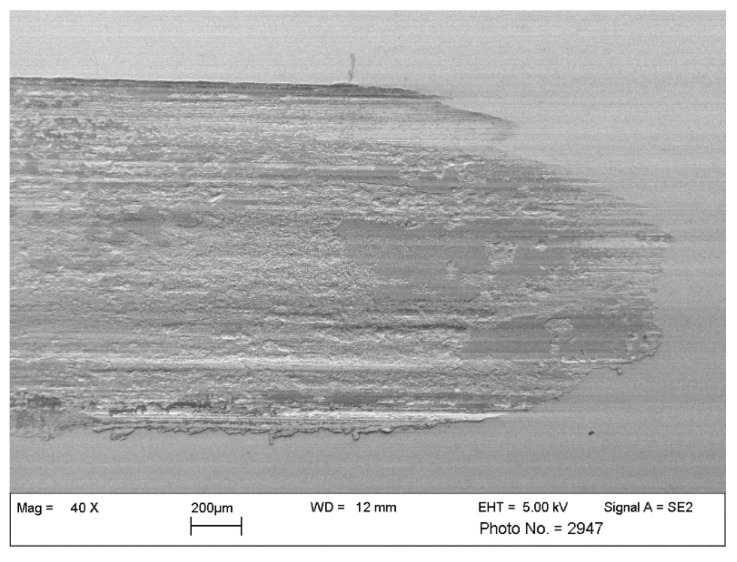
False classification—“surface fatigue”. An overview of the turning point of a sliding wear track is displayed at low magnification. A high number of delaminations are recognizable by the expert but are typically supported by additional, high magnification images.

In the following, an incorrect classification of an image showing the wear mechanism surface fatigue will be considered. The artificial neural network classifies this image to the class “other wear mechanisms”, although it belongs to the class “surface fatigue”. The respective image is shown in Figure 11. One possible reason for the incorrect classification is the magnification of this SEM image, since only a 40-fold magnification has been used. In practice, the recording of such an overview image is accompanied by a high number of further, high magnification images of the same surface region, which display the wear appearances more clearly to the expert.

Within the training dataset, only four images with such a low magnification factor are present. Therefore, images based on a magnification of this magnitude are severely under-represented within the training of the artificial neural network. Accordingly, reduced classification accuracy is obtained for these images. A possibility to counteract this issue is again the extension of the database, with the focus on a homogeneity within the classes concerning the magnification, but also further influencing factors including the contrast and brightness, the selected SEM detectors, or the dimensions of the features of wear appearances in relation to the recorded field of view. For future experimental studies, it must be considered that the interpretation of SEM images should be conducted in context to each other; the comparison of different magnifications and different regions on a certain sample are methods typically used by experts but are not suitable for potential future image analysis by artificial intelligence. Still, the way SEM images are recorded can easily be adapted to the requirements of an AI, if understood by the SEM operator.

## 5. Conclusions

The understanding of predominant wear mechanisms is an important factor with regard to the determination of components’ lifetimes or changes in performance. For this purpose, it is necessary to first identify the mechanism in order to be able to initiate targeted countermeasures. Scanning electron microscope images, which are evaluated by experts through professional knowledge, often serve as a basis for the identification. In this contribution, on the other hand, a detection and classification of the wear mechanisms was implemented by the use of artificial intelligence. The application is based on a binary classification task, where the wear mechanism surface fatigue is distinguished from other wear mechanisms. The database comprises 778 images, which have been extended to 1072 images by oversampling with respect to a class imbalance. For the implementation of the artificial neural network, first, a hyperparameter optimization was performed before the final training was carried out based on the determined parameters. The classification by the artificial neural network results in an accuracy of about 98% for the training data, about 72% for the validation data, and about 73% for the test data.

In conclusion, it has been shown that the complex task of classifying wear mechanisms can be successfully achieved by using artificial intelligence. Further exploitation of the untapped potential of artificial intelligence in this context will be pursued in future research. Thereby, the extension of the classification task with respect to further wear mechanisms, such as adhesion, as well as the enlargement of the database will be the focus of future research, with the objective to provide a homogeneous and balanced database. Furthermore, the adaptation of the expert approach regarding wear mechanism detection for artificial-intelligence-based implementation will be investigated, where coherent images of one sample with different magnifications will be considered in context.

## Figures and Tables

**Table 1:** Chemical composition of tested materials in wt. %.

Material	Al	C	Cr	Cu	Mo	Ni	Mn	P	V	S	Si	Ti	Fe
ER 7	-	0.52	0.3	0.3	0.08	0.3	0.8	0.02	0.06	0.015	0.4	-	bal.
CP4M	-	0.6	5	-	-	-	-	-	-	-	-	-	bal.
Cr60Ni40	<0.25	<0.1	58–60	-	-	bal.	<1	<0.2	-	<0.2	<1	<0.5	<1
100Cr6	-	1	1.5	-	-	-	0.35	-	-	-	0.25	-	bal.

**Table 2:** Experimental conditions for sliding wear tests.

Base Body	Counter Body	Cycles	Normal Force in N	Lubricant
ER7	100Cr6	25,000	20	Silicon oil 50cSt
			
CP4M		500,000–1,000,000	60–180	Anticorit PL3802 39S (60 °C)
				Silicon oil 50cSt
			
Cr60Ni40		12,500–25,000	40–60	Silicon oil 50cSt

**Table 3:** Database.

Process	Surface Fatigue	Surface Fatigue Augmented	Other Wear Mechanisms
Training images	170	384	384
Validation images	36	76	76
Testing images	36	76	76

**Table 4:** Fixed hyperparameters.

Parameter	Value
Batch size	16
Training epochs	25
Optimization iterations	25
Metric	Binary cross entropy
Optimizer	Adam
Layer	Convolutional

**Table 5:** Search space of the hyperparameter optimization.

Parameter	Range	
Filter size	16, 32, 64	
Kernel size	2 × 2	6 × 6
Learning rate	0.0001	0.1
Number of convolutional layers	1	4

**Table 6:** Hyperparameter configuration—results of the hyperparameter optimization.

Parameter	Value
Number of convolutional layers	3
Filter size of the first and third layer	64
Filter size of the second layer	32
Kernel size of the first layer	2 × 2
Kernel size of the second and third layer	6 × 6
Learning rate	0.0038

## Data Availability

Not applicable.
